# A large proportion of poor birth outcomes among Aboriginal Western Australians are attributable to smoking, alcohol and substance misuse, and assault

**DOI:** 10.1186/s12884-019-2252-4

**Published:** 2019-04-02

**Authors:** Alison J. Gibberd, Judy M. Simpson, Jocelyn Jones, Robyn Williams, Fiona Stanley, Sandra J. Eades

**Affiliations:** 1 Faculty of Medicine and Health, Sydney School of Public Health, The University of Sydney, Sydney, New South Wales Australia; 2 Faculty of Health and Medical Sciences, The University of Western Australia, Perth, Western Australia Australia; 3 Baker Heart and Diabetes Institute, Aboriginal Health Domain, Melbourne, Victoria Australia; 4 Telethon Kids Institute, The University of Western Australia, Perth, Western Australia Australia; 5 Melbourne School of Population and Global Health, The University of Melbourne, Level 3, 207 Bouverie St, Melbourne, Victoria 3010 Australia

**Keywords:** Birthweight, Preterm birth, Perinatal mortality, Aboriginal and Torres Strait islander Australians, Indigenous, Linked routinely-collected data

## Abstract

**Background:**

Aboriginal infants have poorer birth outcomes than non-Aboriginal infants. Harmful use of tobacco, alcohol, and other substances is higher among Aboriginal women, as is violence, due to factors such as intergenerational trauma and poverty. We estimated the proportion of small for gestational age (SGA) births, preterm births, and perinatal deaths that could be attributed to these risks.

**Methods:**

Birth, hospital, mental health, and death records for Aboriginal singleton infants born in Western Australia from 1998 to 2010 and their parents were linked. Using logistic regression with a generalized estimating equation approach, associations with birth outcomes and population attributable fractions were estimated after adjusting for demographic factors and maternal health during pregnancy.

**Results:**

Of 28,119 births, 16% of infants were SGA, 13% were preterm, and 2% died perinatally. 51% of infants were exposed in utero to at least one of the risk factors and the fractions attributable to them were 37% (SGA), 16% (preterm) and 20% (perinatal death).

**Conclusions:**

A large proportion of adverse outcomes were attributable to the modifiable risk factors of substance use and assault. Significant improvements in Aboriginal perinatal health are likely to follow reductions in these risk factors. These results highlight the importance of identifying and implementing risk reduction measures which are effective in, and supported by, Aboriginal women, families, and communities.

**Electronic supplementary material:**

The online version of this article (10.1186/s12884-019-2252-4) contains supplementary material, which is available to authorized users.

## Background

Australian Aboriginal and Torres Strait Islander (hereafter respectfully referred to as Aboriginal) infants tend to have poorer birth outcomes than non-Aboriginal infants. In the state of Western Australia (WA), preterm birth, stillbirth, and neonatal death rates are 2–3 times higher and the average birthweight is 200 g less for infants with Aboriginal mothers than non-Aboriginal mothers [1, 2]. In the past three decades, though the neonatal death rate has declined, the rates of small for gestational age (SGA) births, preterm births and stillbirths to Aboriginal mothers in WA have remained static [1–3] and a better understanding of why these outcomes are so common among Aboriginal infants is needed.

Smoking during pregnancy, harmful use of alcohol and drugs, and assault against the mother are all associated with poor birth outcomes [4, 5] and are also more common among Aboriginal than non-Aboriginal women. The context in which they arise is generations of displacement from traditional lands, limited education and employment opportunities resulting in economic disadvantage, marginalization, racism, forced removal of children from their parents, and other associated losses.

Aboriginal women smoke during approximately half of all pregnancies [6]. While abstinence from alcohol is common in Aboriginal communities, among those who do drink, consumption is more likely to be harmful and Aboriginal women are seven times more likely to die from alcoholic liver cirrhosis and alcohol dependence than non-Aboriginal women [7]. Rates of drug use are also high. In 2015, 27% of Aboriginal women reported using substances in the previous 12 months for non-medical reasons [8]. By contrast, 13% of non-Aboriginal women reported illicit drug use [9]. Finally, Aboriginal women are 35 times more likely to be hospitalised because of an assault than non-Aboriginal women [10]. The use of tobacco, alcohol and drugs, and assault are inter-related and multiple risk factors can aggregate in pregnancy [11].

The associations of poor birth outcomes with smoking, alcohol, drugs, and assault have been observed across a range of populations [4, 5]. However, their contribution to the high levels of poor birth outcomes among Aboriginal infants is rarely quantified, particularly for assault. We therefore aimed to estimate the proportions of Aboriginal SGA births, preterm births, and perinatal deaths in WA from 1998 to 2010 that can be attributed to smoking, misuse of alcohol or drugs, and assault.

## Methods

### Study cohort and data sources

The study cohort comprised all singleton births in WA from 1998 to 2010, where the infant and their full siblings were categorised as Aboriginal using the algorithm MSM + Family, described in Gibberd et al [12]. Briefly, this algorithm assigns Aboriginal status to each infant using the Indigenous identifiers on their birth record (Midwives Notification System [MNS]), birth registration, inpatient hospital records (Hospital Morbidity Data Collection), and WA Register of Developmental Anomalies (WARDA) record, as well as their family members’ records. The algorithm offers some protection against false positives that can occur with linkage of many records, while relatives’ information resolves some false negatives and positives and reduces the number of infants with unknown Aboriginal status [12]. The study cohort’s relatives were identified by WA’s records of family links, the Family Connections System [13].

The Data Linkage Branch in the Department of Health linked the above datasets and death records using probabilistic linkage.

In total, 28,119 Aboriginal infants were recorded in the MNS from 1998 to 2010. Each birth with a gestational age of at least 20 weeks and/or a birthweight of 400 g is notified to the WA Department of Health by an attending midwife or medical officer. Details of the mother and infant, the birth, and conditions affecting the mother or pregnancy are recorded on the birth record.

#### Outcomes

The three outcomes of interest were SGA, preterm birth, and perinatal death. An infant was defined as SGA if their birthweight was less than the first decile for Australian singleton infants of the same sex and gestational age, born alive from 1998 to 2007 [14]. Preterm birth was defined as any live birth or stillbirth at 20 to 36 completed weeks’ gestation. In line with the Australian policy of classification of perinatal deaths, they were defined as either stillbirth (the death of a baby prior to the complete expulsion or extraction from its mother at a gestational age of 20 or more completed weeks or with a birthweight of at least 400 g) or death less than 28 days after a live birth [15].

Gestational age (GA) as determined by Blair et al’s method [16] was missing for 67 of the 28,119 infants. All 67 infants had an estimated gestational age of 20 to 34 weeks in their birth records, which was based on observations of the neonate, including sole creases and scalp hair. We classified all 67 infants as preterm because, even if this estimate was less accurate than Blair et al’s method, the magnitude of the error would need to be at least 3 weeks for an infant to be misclassified as preterm. However, as classification as SGA was based on actual GA, infants with missing GA were excluded from analyses involving SGA.

#### Study factors

The four risk factors of interest were maternal smoking, alcohol misuse, drug misuse, and assault during pregnancy. Maternal smoking has been recorded comprehensively on WA birth records since 1998. Mothers were categorised as misusing alcohol or drugs if an alcohol- or drug-related diagnosis was recorded in (1) the child’s birth record or (2) in any diagnosis field on their hospital admissions during the pregnancy or (3) a mental health record during the pregnancy.

The mother was categorised as the victim of assault if violence against her was recorded in a hospital admission in the period from two years prior to the start of the pregnancy until the birth. We included this ‘look-back’ period because around half of all women who suffer physical violence before pregnancy continue to be exposed during pregnancy and socio-economically deprived women are particularly likely to have violence continue into pregnancy [17], violence prior to pregnancy is an independent predictor of poor birth outcomes [18], and a look-back period was likely to improve ascertainment of cases of assault as we could only identify violence through external injury codes in one dataset. We did not include a look-back period for alcohol or drug misuse as we had additional data sources to identify misuse and the vast majority of women cease or reduce their consumption during pregnancy [19, 20]. These issues did not arise for smoking, a mandatory field in the birth record.

#### Other explanatory variables

We initially chose explanatory variables that are known, or suspected, to be associated with the birth outcomes, were recorded in our data, and were not rare. They were *demographic information* (infant year of birth, infant sex, maternal parity, and maternal age), *maternal infections during pregnancy* (urinary tract infections [UTI], *Herpes simplex*, gonorrhoea, chlamydia, vaginitis [vaginitis, *Candida*, and/or trichomoniasis], Group B streptococcus, and other infections [syphilis, toxoplasmosis, rubella, cytomegalovirus, and/or varicella zoster]), and *maternal long-term health* (maternal height, diabetes, hypertension, obesity, mental health conditions, heart disease, and asthma).

We identified maternal health conditions from both the infant’s birth record and the mother’s hospital records during pregnancy as the combination improves ascertainment with little change in the number of false positives [21]. Using broad categories for diseases during pregnancy also increases sensitivity, with minimal change in specificity [21]. “Diabetes” included pre-existing and gestational diabetes, and “hypertension” included pre-existing hypertension complicating pregnancy, pre-eclampsia, and eclampsia. All relevant codes of the International Classification of Diseases, 9th and 10th Revisions (ICD-9-CM, ICD-10-AM) are listed in Additional file 1.

#### Analysis

In the adjusted logistic regression model for each outcome, we initially included the study factors (maternal smoking, alcohol misuse, drug misuse, assault) and all other explanatory variables with *P* < 0.2 in unadjusted models. Variables were sequentially removed until only variables with *P* < 0.05 and the study factors remained. We then entered interactions with the study factors, except interactions with maternal height which we did not believe were biologically plausible. With no prior reason to believe there would be interactions, we set the significance level at 0.01. We then entered all significant interactions into the model simultaneously and retained those that remained significant. We then checked the variable selection by adding the excluded variables to the model again, one-by-one. However, they remained non-significant and their inclusion did not meaningfully change the coefficients for the four study factors.

We used the multivariable fractional polynomial procedure to test whether non-linear functional forms for the continuous variables were preferable [22]. In the fully adjusted models, maternal height had linear associations with all three outcomes and infant’s year of birth had linear or no association. Transformations of maternal age of degree 1 and degree 3 were selected for the preterm birth model and degree -½ and degree 3 for the perinatal death model.

Maternal height, an important predictor of birth outcomes [23], was missing for 9305/28119 (33%) of births. However, for 6078/9305 cases, maternal height was available in siblings’ birth records. We used multiple imputation to impute the remaining 3227 (11%) missing cases (Additional file 1) [24]. We created 20 complete datasets.

Regression coefficients and variances were obtained from models fit to each of the 20 datasets using logistic regression with a generalised estimating equation (GEE) approach to account for correlation within mothers. Independent working correlation matrices and robust standard errors were selected. Using Rubin’s rules, we combined the 20 sets of regression coefficients and variances [25].

Parents may have children with more than one partner and those partners may also have children with more than one partner. As a result, children are not all clustered in nuclear families and can be cross-classified to mothers and fathers. We calculated regression coefficient covariance matrices that took cross-classification into account (Additional file 1). Because these matrices were very similar to those obtained by clustering on the mother, we present the results from clustering by mother only.

Population attributable fractions (PAFs) are the proportions of disease attributable to an exposure or group of exposures. We calculated model-based adjusted PAFs for the risk factors of interest by calculating the difference between the observed number of poor outcomes and the expected number if the risk factor was eliminated from the population, divided by the observed number of outcomes [26]. We estimated 95% confidence intervals using bootstrap with 1000 replicates.

SAS software, Version 9.4, was used for all analyses, with some exceptions. R 3.4.0 [27] was used for the multiple imputations, to identify appropriate fractional polynomials, to obtain bootstrap samples, and to calculate population attributable fractions (using regression coefficients obtained using SAS).

#### Sensitivity and subgroup analyses

We conducted sensitivity analyses by analysing the subset of 18,814 out of 28,119 births which had maternal height recorded on their birth records (‘complete cases’). As we did not include remoteness or socioeconomic disadvantage to avoid overfitting, sensitivity analyses were also conducted with these variables included. Finally, as research often focuses on first-born infants and birth weight varies by parity, we also stratified by parity.

## Results

Approximately a quarter (27%) of the 28,119 infants had at least one of the three outcomes of interest: 16% of infants were SGA; 13% of infants were preterm; and 2% died perinatally (Table 1). Mothers smoked during 47% of the pregnancies and alcohol misuse was recorded for 3% of pregnancies, drug misuse for 6%, and assault for 7%. For 51% of births, at least one of these risks was present.

**Table 1:** Associations between birth outcomes and infant and maternal characteristics for 28,119 WA Aboriginal singletons born 1998–2010

Characteristic		Overall (column %)	Small for gestational age (row %)		Preterm birth (row %)		Perinatal death (row %)	
			Yes	No	Yes	No	Yes	No
Risk factors of interest								
Maternal smoking	Yes	13,292 (47)	2821 (21)	10,435 (79)	1974 (15)	11,318 (85)	267 (2)	13,025 (98)
	No	14,827 (53)	1584 (11)	13,212 (89)	1679 (11)	13,148 (89)	197 (1)	14,630 (99)
Alcohol misuse	Yes	799 (3)	286 (36)	509 (64)	187 (23)	612 (77)	27 (3)	772 (97)
	No	27,320 (97)	4119 (15)	23,138 (85)	3466 (13)	23,854 (87)	437 (2)	26,883 (98)
Drug misuse	Yes	1824 (6)	488 (27)	1334 (73)	454 (25)	1370 (75)	38 (2)	1786 (98)
	No	26,295 (94)	3917 (15)	22,313 (85)	3199 (12)	23,096 (88)	426 (2)	25,869 (98)
Assault against mother	Yes	2015 (7)	523 (26)	1482 (74)	395 (20)	1620 (80)	37 (2)	1978 (98)
	No	26,104 (93)	3882 (15)	22,165 (85)	3258 (12)	22,846 (88)	427 (2)	25,677 (98)
Demographic factors								
Infant’s sex	Male	14,161 (50)	2304 (16)	11,824 (84)	1865 (13)	12,296 (87)	241 (2)	13,920 (98)
	Female	13,958 (50)	2101 (15)	11,823 (85)	1788 (13)	12,170 (87)	223 (2)	13,735 (98)
Maternal age (years)	12–15	556 (2)	93 (17)	457 (83)	89 (16)	467 (84)	16 (3)	540 (97)
	16–19	5717 (20)	1077 (19)	4621 (81)	750 (13)	4967 (87)	91 (2)	5626 (98)
	20–24	9008 (32)	1403 (16)	7584 (84)	1144 (13)	7864 (87)	138 (2)	8870 (98)
	25–29	6816 (24)	973 (14)	5832 (86)	827 (12)	5989 (88)	111 (2)	6705 (98)
	30–34	3994 (14)	574 (14)	3412 (86)	532 (13)	3462 (87)	62 (2)	3932 (98)
	35–50	2028 (7)	285 (14)	1741 (86)	311 (15)	1717 (85)	46 (2)	1982 (98)
Parity	0	8636 (31)	1634 (19)	6979 (81)	1034 (12)	7602 (88)	138 (2)	8498 (98)
	1	6898 (25)	973 (14)	5911 (86)	858 (12)	6040 (88)	100 (1)	6798 (99)
	2 or more	12,585 (45)	1798 (14)	10,757 (86)	1761 (14)	10,824 (86)	226 (2)	12,359 (98)
Infections during pregnancy								
Vaginitis	Yes	1747 (6)	305 (17)	1439 (83)	464 (27)	1283 (73)	43 (2)	1704 (98)
	No	26,372 (94)	4100 (16)	22,208 (84)	3189 (12)	23,183 (88)	421 (2)	25,951 (98)
Urinary tract infection	Yes	3997 (14)	720 (18)	3269 (82)	639 (16)	3358 (84)	89 (2)	3908 (98)
	No	24,122 (86)	3685 (15)	20,378 (85)	3014 (12)	21,108 (88)	375 (2)	23,747 (98)
Herpes simplex	Yes	320 (1)	33 (10)	286 (90)	42 (13)	278 (87)	6 (2)	314 (98)
	No	27,799 (99)	4372 (16)	23,361 (84)	3611 (13)	24,188 (87)	458 (2)	27,341 (98)
Gonorrhoea	Yes	192 (1)	56 (30)	133 (70)	44 (23)	148 (77)	n.p.	n.p.
	No	27,927 (99)	4349 (16)	23,514 (84)	3609 (13)	24,318 (87)	n.p.	n.p.
Chlamydia	Yes	255 (1)	47 (21)	177 (79)	44 (20)	181 (80)	5 (2)	220 (98)
	No	27,894 (99)	4358 (16)	23,470 (84)	3609 (13)	24,285 (87)	459 (2)	27,435 (98)
Group B streptococcus	Yes	1333 (5)	208 (16)	1124 (84)	256 (19)	1077 (81)	22 (2)	1311 (98)
	No	26,786 (95)	4197 (16)	22,523 (84)	3397 (13)	23,389 (87)	442 (2)	26,344 (98)
Other infections	Yes	148 (1)	40 (27)	107 (73)	26 (18)	122 (82)	6 (4)	142 (96)
	No	27,971 (99)	4365 (16)	23,540 (84)	3627 (13)	24,344 (87)	458 (2)	27,513 (98)
Other maternal conditions								
Diabetes	Yes	1841 (7)	151 (8)	1688 (92)	373 (20)	1468 (80)	46 (2)	1795 (98)
	No	26,278 (93)	4254 (16)	21,959 (84)	3280 (12)	22,998 (88)	418 (2)	25,860 (98)
Hypertension	Yes	2811 (10)	503 (18)	2303 (82)	579 (21)	2232 (79)	45 (2)	2766 (98)
	No	25,308 (90)	3902 (15)	21,344 (85)	3074 (12)	22,234 (88)	419 (2)	24,889 (98)
Obesity	Yes	600 (2)	39 (7)	559 (93)	116 (19)	484 (81)	13 (2)	587 (98)
	No	27,519 (98)	4366 (16)	23,088 (84)	3537 (13)	23,982 (87)	451 (2)	27,068 (98)
Mental health	Yes	2017 (7)	328 (16)	1686 (84)	357 (18)	1660 (82)	47 (2)	1970 (98)
	No	26,102 (93)	4077 (16)	21,961 (84)	3296 (13)	22,806 (87)	417 (2)	25,685 (98)
Heart disease	Yes	225 (1)	35 (16)	190 (84)	48 (21)	177 (79)	5 (2)	220 (98)
	No	27,894 (99)	4370 (16)	23,457 (84)	3605 (13)	24,289 (87)	459 (2)	27,435 (98)
Asthma	Yes	3136 (11)	477 (15)	2657 (85)	424 (14)	2712 (86)	42 (1)	3094 (99)
	No	24,983 (89)	3928 (16)	20,990 (84)	3229 (13)	21,754 (87)	422 (2)	24,561 (98)
Total		28,119 (100)	4405 (16)	23,647 (84)	3653 (13)	24,466 (87)	464 (2)	27,655 (98)

n.p. = counts not publishable because of privacy concerns as they are less than 5 or could lead to calculation of a count of less than 5. Hypertension refers to pre-existing hypertension complicating pregnancy, pre-eclampsia, and eclampsia. Vaginitis also includes candida and trichomoniasis. Other infections refer to syphilis, toxoplasmosis, rubella, cytomegalovirus, and varicella zoster. ^a^ 67 cases of unknown gestational age were excluded

**Fig. 1:**
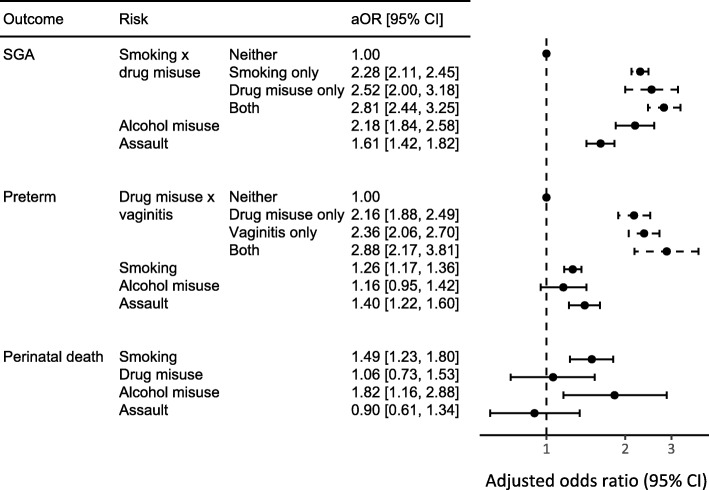
Adjusted odds ratios (aOR) of birth outcomes from smoking, alcohol misuse, drug misuse, and assault. Adjusted odds ratios are for 28,119 Aboriginal singleton infants born in Western Australia, 1998–2010. Bars are 95% confidence intervals. Each model adjusted for maternal smoking, drug misuse, alcohol misuse, assault, maternal height and diabetes. The model for SGA also included an interaction between maternal smoking and drug misuse, infant sex, parity, hypertension (pre-existing hypertension complicating pregnancy, pre-eclampsia, and eclampsia), obesity, gonorrhoea, herpes, and other infections (syphilis, toxoplasmosis, rubella, cytomegalovirus, and varicella zoster). The model for preterm birth also included an interaction between drug misuse and vaginitis (vaginitis, candida and trichomoniasis), maternal age, parity, infant’s year of birth, hypertension, heart disease, urinary tract infection, Group B streptococcus, obesity, mental health conditions, and gonorrhoea. The model for perinatal death also included maternal age and urinary tract infection. Confidence intervals are dashed for risks with interactions and solid otherwise

Maternal smoking was associated with over twice the odds of SGA birth, 26% higher odds of preterm birth and 49% higher odds of perinatal death (Fig. 1). Alcohol was associated with 118% higher odds of SGA and 83% high odds of perinatal death, but the association with preterm birth, while positive, was not statistically significant. Drug misuse and assault were strongly associated with SGA and preterm birth, but not perinatal death.

There were two interactions with the risk factors of interest. Compared to mothers who neither smoked nor misused drugs, those who either smoked or misused drugs had over twice the odds of a SGA infant (adjusted odds ratio (aOR) 2.28 [95% CI: 2.12, 2.46] for smoking only and 2.52 [95% CI: 2.00, 3.19] for drug misuse only). However, if the mother both smoked and misused drugs, the infant’s odds of being SGA were not much greater (aOR 2.82 [95% CI: 2.44, 3.25]). Similarly, for preterm birth, there was an interaction between drug misuse and vaginitis.

**Fig. 2:**
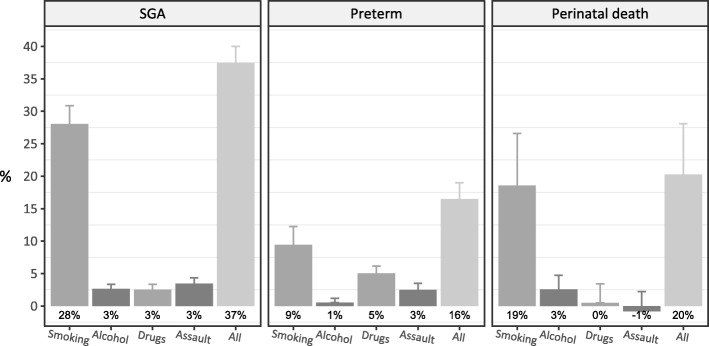
Adjusted population attributable fractions for birth outcomes from smoking, alcohol misuse, drug misuse, and assault. Adjusted population attributable fractions with 95% confidence intervals are for 28,119 Aboriginal singleton infants born in Western Australia, 1998–2010. Each model adjusted for maternal smoking, drug misuse, alcohol misuse, assault, maternal height and diabetes. The model for SGA also included an interaction between maternal smoking and drug misuse, infant sex, parity, hypertension (pre-existing hypertension complicating pregnancy, pre-eclampsia, and eclampsia), obesity, gonorrhoea, herpes, and other infections (syphilis, toxoplasmosis, rubella, cytomegalovirus, and varicella zoster). The model for preterm birth also included an interaction between drug misuse and vaginitis (vaginitis, candida and trichomoniasis), maternal age, parity, infant’s year of birth, hypertension, heart disease, urinary tract infection, Group B streptococcus, obesity, mental health conditions, and gonorrhoea. The model for perinatal death also included maternal age and urinary tract infection

37% of SGA births, 16% of preterm births and 20% of perinatal deaths could be attributed to smoking, alcohol misuse, drug misuse or assault. As PAFs are affected by prevalence of the risk factor, as well as the magnitude of the risk, smoking had the highest PAF for each outcome (Fig. 2).

Results from analyses of complete cases were similar to the main results using imputed maternal heights, with the exception of perinatal death, where the odds were only 29% higher for alcohol misuse than no misuse among the complete cases, compared to 83% higher among the full sample (Additional file 1: Tables S6 and S8). The addition of remoteness and socioeconomic disadvantage to the models slightly attenuated the significant relationships between the poor birth outcomes and alcohol misuse and assault. However, the odds ratios for smoking changed little and those for drug misuse increased. The PAFs changed little with the inclusion of remoteness and socioeconomic disadvantage (Additional file 1: Table S9).

## Discussion

Between 1998 and 2010, 27% of all Aboriginal infants in WA were SGA, preterm, or died perinatally. A substantial proportion of these outcomes could be attributed to in utero exposure to maternal smoking, alcohol misuse, drug misuse, and assault against their mother -37% of SGA births, 16% of preterm births, and 20% of perinatal deaths. With half (51%) of the infants exposed to at least one of these risk factors, reductions in these interrelated behaviours may greatly improve Aboriginal perinatal health.

More poor outcomes could be attributed to smoking than the other risks, reflecting the fact that 47% of infants were exposed. We found 28% of SGA births could be attributed to smoking. By contrast, Taylor et al found that for infants born to mothers in the state of New South Wales (NSW, 97% non-Aboriginal mothers) with a smoking rate of 11% during pregnancy, only 10% of SGA births for term infants with non-diabetic mothers were attributable to smoking, 3% for term infants with diabetic mothers, and 12% of SGA births for preterm infants [28].

It is likely we underestimated the prevalence of alcohol and drug misuse and assault as this information was not mandatory in the datasets. It is also likely that we identified the more serious cases, given we identified most cases through hospital admissions and the nature of ICD diagnoses. Our estimates may also be lower than other studies as we included Aboriginal infants with non-Aboriginal mothers (18% of births). For example, only 0.5% of infants to non-Aboriginal mothers were categorised as exposed to alcohol, compared to 3.3% of infants with Aboriginal mothers.

The true proportion of infants subject to harmful levels of alcohol in utero is difficult to assess. It is not clear which drinking patterns (timing, frequency, and amount) are harmful and few studies have collected detailed information from Aboriginal women. In WA studies, 23% or more Aboriginal women drank during pregnancy, though this included any alcohol consumption [29], harmful consumption from age 10 to a year after pregnancy [30], or the sample was highly selected, including a community with high average consumption [31, 32].

Our finding that 6% of infants were exposed to drug misuse may be more accurate than our finding about alcohol misuse. WA Aboriginal mothers have reported using marijuana during pregnancy for 9% of births and other drugs for fewer than 1% [29], though some studies from other states have found greater drug use [11, 33].

The proportion of infants whose mothers were categorised as victims of assault (7%) was half the proportion of all Aboriginal women reported by the Australian Bureau of Statistics to have experienced physical violence in the past year in 2014–15 (14%) [34]. Nevertheless, the higher odds of SGA and preterm birth following assault, compared to no assault, (aOR: 1.61 and 1.40, respectively) were similar to those from a 2010 meta-analysis for low birth weight (aOR: 1.53 [95% CI: 1.28, 1.82]) and preterm birth (1.46 [95% CI: 1.27, 1.67]) [4]. Hypotheses about the effect of maternal stress hormones during pregnancy have been proposed, for example, the release of oxytocin could induce early contractions [35].

Although we underestimated the prevalence of some risk factors, as they are often clustered in women the PAF for all risks combined may be a reasonable estimate of the true PAF. For example, if a woman smoked and misused alcohol, but only smoking was documented, her contribution to the risk estimate for smoking may encompass the effects of both smoking and alcohol, resulting in a higher risk estimate for smoking. When the PAF was calculated for all risk factors combined, some of this additional risk from (unidentified) alcohol misuse would be captured in the combined PAF.

Approximately 1% of pregnant Aboriginal women in WA have no antenatal care and 17% have no care until after 24 weeks’ gestation [2]. Antenatal care attendance could not be explored in our analysis as this information was not available for the birth years covered by this study. Substance misuse and assault and suboptimal antenatal care are clearly associated. However, it is not clear whether substance misuse and assault affect attendance for antenatal care or whether they share common causes. If the former, antenatal care is an intermediate variable and including antenatal care in the model might have biased the results. If the latter, and if the common causes are not adjusted for, our estimates of the effects of the risk factors may be biased upwards.

Births following terminations of pregnancy from 20 weeks’ gestation or with congenital abnormalities were not excluded because we were interested in population-level outcomes, substance misuse is associated with certain developmental abnormalities [36], and some of the poor birth outcomes in this study may have followed the combination of developmental abnormalities and the risk factors of interest. The proportion of preventable poor birth outcomes would have been higher in a sample of births which did not include terminations of pregnancy or congenital abnormalities than in the full population, most likely resulting in higher estimates of the PAFs.

For this study we used a composite endpoint of stillbirths and neonatal deaths, as estimates obtained from modelling the 141 neonatal deaths separately would have a high degree of uncertainty, ‘live-birth bias’ may bias our estimates, and because the causes of the majority of neonatal deaths arise prenatally or in the intrapartum period [37, 38].

Smoking, alcohol and drug misuse, and violence are intrinsically linked and may be triggered by boredom, unemployment, marginalisation, poor mental health, overcrowded housing, and other stresses more commonly experienced by Aboriginal people [39–41]. Many Aboriginal people have complex health and social needs and some mainstream initiatives have been less effective in Aboriginal populations. For example, while smoking among pregnant Aboriginal women has dropped considerably, the decrease in recent years has been greater for non-Aboriginal women and non-Aboriginal women are more likely to quit smoking during pregnancy [6].

Aboriginal women can face significant barriers to change. Smoking and violence are normalised in some communities [39, 42] and drinking is frequently social with 27% of 180 Nyoongar women reporting drinking with a male partner while pregnant (Robyn Williams, personal communication, 7 November 2017). Fear of losing children to child protection agencies can discourage women from seeking help with substance misuse and violence [40]. WA Aboriginal children are 17.5 times more likely to be in out-of-home care than non-Aboriginal children [43]. Numerous additional challenges may affect Aboriginal women, such as limited services [40, 41].

Despite the widespread acknowledgement that Aboriginal-specific risk reduction measures are needed, rigorous evaluations of Aboriginal-specific responses are rare [44–46]. To the best of our knowledge only one randomised clinical trial involving pregnant Aboriginal women has been conducted. This trial aimed to assess the effect of a smoking cessation intervention which included advice about quitting smoking at a woman’s first antenatal appointment, follow-up appointments with Aboriginal healthcare workers and midwives, and nicotine replacement therapy [47]. More women in the treatment (psychosocial) arm quit smoking than in the standard care arm, but the difference was not statistically significant. The trial faced difficulties with high staff turnover, possible contamination between the two arms, and over 30% loss to follow-up with only 176 completing. Methodologically rigorous studies can be more difficult in Indigenous populations, with challenges such as small sample sizes and funding time-limits may be insufficient to establish and maintain relationships with communities [48]. Funding incentives and alternative governance approaches may encourage more studies [48].

Evaluations of restrictions (including bans) on the supply of alcohol to communities have found that, with Aboriginal leadership and community support, they can be effective in reducing consumption and related harms like violence, despite some unintended consequences [49]. The 2018 decision to introduce a minimum price for a unit of alcohol across the NT may provide evidence of the impact of price signals [50].

The lack of evidence about how to prevent violence against Aboriginal women is unsurprising as, globally, little is known about what works [51]. Some Aboriginal communities have night patrols. Evidence of efficacy is limited but many patrols are valued by community members, local police, and service providers, suggesting they have a positive impact [40].

Relevant and accessible data are essential to improve the evidence base and calls have recently been made for increased data collection and linkage; for example, the establishment of a national data collection on violence and the expansion of perinatal data collections to include details of domestic violence and substance use [52, 53]. In WA, from 2017, the quantity and frequency of alcohol consumption during pregnancy will be available. Routinely-collected data can be a cost-effective way of evaluating programs. Outcomes can be passively measured at many time points, loss to follow up due to relocation within the state is minimized, and data collection may be more objective. With population-based data, robust estimates of the scale of these issues can be obtained, as in this study.

While the evidence-base for Aboriginal-specific risk reduction measures is limited, studies in other populations have identified effective approaches that could be tailored to Aboriginal communities.

An empirical evidence base is only one possible influence on health policy [46]. Evidence from other populations, studies of the acceptability and feasibility of interventions in an Aboriginal context, and the knowledge of community members and other stakeholders can inform risk reduction policies. There is widespread agreement that programs must be genuine partnerships or Aboriginal-led, tailored to local communities, holistic, targeted at the family and community level, as well as individual, and adequately supported [42, 54, 55]. A supportive, rather than punitive, approach towards Aboriginal women struggling with substance misuse and violence is needed.

Underlying smoking, alcohol and drug use, and violence in Aboriginal communities is a post-colonial history of dispossession, intergenerational trauma, structural racism, and poverty [41, 56]. Addressing the social determinants of these risk factors and poor mental health is an essential part of reducing these risks.

## Conclusions

With half of WA’s Aboriginal infants exposed in utero to the preventable risk factors of smoking, alcohol or drug misuse, or assault against the mother and a large proportion of poor birth outcomes attributable to this exposure, great improvements in the health of Aboriginal babies are possible with reductions in these risk factors. These results highlight the importance of identifying and implementing risk reduction measures which are effective in, and supported by, Aboriginal women, families and communities.

## Additional file

Additional file 1: Additional file contains codes of the International Classification of Diseases, 9th and 10th Revisions (ICD-9-CM, ICD-10-AM) used in this study, additional details of the methodology and additional results. (DOCX 66 kb)
